# Application of flipped classroom in surgical education: a systematic review and meta-analysis

**DOI:** 10.3389/fmed.2026.1841948

**Published:** 2026-05-28

**Authors:** Rui Cheng, Sike He, Yuan Wang, Huan Ren, Xingming Zhang, Yunjin Bai

**Affiliations:** 1 West China School of Medicine, Sichuan University, Chengdu, China; 2 Department of Urology, Institute of Urology, West China Hospital, Chengdu, China

**Keywords:** flipped classroom, medical education, meta-analysis, surgical education, traditional lecture

## Abstract

**Background:**

Surgical education is crucial to patient care as it trains surgeons and equips medical students with knowledge and skills. Flipped classroom (FC) is an active teaching approach that has shown potential in medical education, but its impact on surgical education remains unclear. This study aimed to evaluate the effectiveness of FC compared with traditional lecture (TL) in surgical education.

**Methods:**

Web of Science, PubMed, Embase, Cochrane Library, and ClinicalTrials.gov were searched following a predefined protocol. Risk of bias was assessed by the Cochrane Risk of Bias Assessment Tool (version 2) and the “Risk of Bias” tool from the Cochrane Effective Practice and Organization of Care Group. The certainty of evidence (CoE) was measured applying the Grading of Recommendations, Assessment, Development, and Evaluations (GRADE) system. Pooled effects of FC over TL were calculated using standard mean differences (SMDs) and 95% confidence intervals (CIs). Subgroup analyses were performed to explore potential sources of heterogeneity.

**Results:**

A total of 11 studies were included in the systematic review and nine in the meta-analysis. FC showed significant benefits over TL in improving learning outcomes as measured by total scores (SMD = 0.37, 95% CI: 0.11–0.63, *p* = 0.005), theoretical scores (SMD = 0.32, 95% CI: 0.09–0.54, *p* = 0.005), and operational scores (SMD = 0.56, 95% CI: 0.33–0.78, *p* < 0.00001). Study design, study direction, continent, curriculum type, and exam type may affect heterogeneity. The GRADE showed high CoE for total and operational scores and moderate CoE for theoretical scores in randomized studies, but very low CoE for three outcomes in non-randomized studies.

**Conclusion:**

Current evidence suggests that FC may be more effective in surgical education for improving students’ academic performance compared with TL. It may serve as a promising approach for surgical education. More high-quality studies are expected to confirm these findings.

**Systematic review registration:**

https://www.crd.york.ac.uk/PROSPERO/view/CRD42024602869, identifier PROSPERO (CRD42024602869).

## 1 Introduction

Surgery is a vital discipline in medicine, significantly contributing to patient survival and functional recovery across a wide range of conditions. Surgical education is crucial to patient care as it not only trains surgeons and enhances their performance but also equips medical students and interns with essential theoretical knowledge, clinical reasoning abilities, and technical skills (1, 2). As healthcare continues to evolve, strengthening surgical education remains crucial for addressing current and future clinical challenges and for sustaining high standards of medical practice (3).

The primary teaching approach for surgical education is traditional lecture (TL), which is teacher-centered with a strong emphasis on theoretical instruction and lacks interaction. TL is considered a passive teaching model, causing students’ disengagement and reducing their learning efficacy (4). Moreover, students’ attention drops in TL and they are unable to recall most of the class material, reducing effectiveness of the class (5, 6).

Flipped classroom (FC, also known as flipped learning) originally introduced by Baker, is an active, student-centered approach that is designed to enhance the effectiveness of in-class learning (7). In the FC model, students are expected to engage in self-directed study of multimedia lectures and other materials (e.g., videos, PowerPoint slides, notes) prior to class, while classroom sessions are dedicated to interactive, student-centered learning activities (8). FC enables students to self-regulate their learning, deepen their understanding during class time, and develop skills in applying newly acquired knowledge. Instead of passively receiving knowledge, FC requires students to explore and interact with peers, teachers, and course materials, creating opportunities for active, effective learning (7). FC has grown increasingly common and is frequently required as part of medical curricula, driven by medical education reform over many years. This trend is particularly evident in disciplines such as nursing education and selected clinical specialties, where FC has been integrated into multiple courses as a standard teaching modality (9–11).

In recent years, growing evidence has demonstrated that FC improves medical students’ academic performance and learning satisfaction in various subjects. A meta-analysis including 11 trials showed that FC had a significant effect on enhancing students’ theoretical knowledge scores and skill scores compared to TL in nursing education (12). Another meta-analysis revealed better academic performance and higher satisfaction of FC in health professional education (11). Additionally, a randomized controlled trial (RCT) reported that the FC group obtained higher exam scores and gave positive feedback to FC in ophthalmology (13). These findings reinforce the effectiveness of FC and suggest that additional studies are needed to examine their relevance in other fields of medical education.

Surgical education requires hands-on practice, clinical observation, and collaboration, which align closely with the features of FC. Despite these potential advantages, the effects of FC in surgical education have not been fully elucidated. Only a limited number of individual studies have examined its impact in contexts such as surgery clerkship and neurosurgery training (14, 15). However, the findings from these studies have sometimes been inconsistent, resulting in inconclusive evidence regarding its effectiveness. To address this gap, our meta-analysis aims to systematically evaluate the effectiveness of FC over TL in surgical education and to provide further insights into its potential role in improving surgical training.

## 2 Materials and methods

This systematic review and meta-analysis was conducted adhering to the Preferred Reporting Items for Systematic Reviews and Meta-Analyses (PRISMA) guidelines (16), Assessing the Methodological Quality of Systematic Reviews (AMSTAR) guidelines (17). The protocol has been registered in the International Registry of Prospective Systematic Reviews (PROSPERO) (https://www.crd.york.ac.uk/prospero, CRD42024602869), and the systematic review was conducted consistently with the registered protocol.

### 2.1 Search strategy

Two reviewers independently searched five electronic databases, including Web of Science, PubMed, Embase, Cochrane Library, and ClinicalTrials.gov, from inception to 30 September 2025. Retrieval strategy: We used the combination of items related to FC (e.g., flipped classroom OR flipped class OR inverted classroom) and surgical education (e.g., surgery OR surgical OR general surgery OR urology). See the full strategy in Supplementary Table 1.

### 2.2 Study selection

Studies were included according to the following criteria: (1) RCT, quasi-experimental study and cohort study; (2) students receiving surgical education, regardless of age, gender, ethnicity, nationality, discipline, and major; (3) the FC approach and the TL approach with clear definition or description were used for group teaching respectively, and the teaching effect was compared; (4) the indicators of the outcomes are measurable data.

Studies were excluded according to the following criteria: (1) duplicated studies; (2) non-original studies, including review, systematic review and meta-analysis, letter to the editor, meeting abstract, expert opinions; (3) lack of the control group; (4) the definition or description for FC and TL was unclear; (5) no direct outcomes or the outcomes were not measurable.

Two researchers completed the literature selection, and the third researcher was responsible for resolving the disagreement.

### 2.3 Data extraction

The following data were extracted from included studies: (a) first author; (b) publication year; (c) country; (d) study type; (e) subjects; (f) participant description; (g) sample size; (h) information on the FC implementation and the TL control; (i) study duration; (j) outcomes: the primary outcome was students’ academic performance which was categorized into total score, theoretical score, and operational score considering the specific features of surgical education; the secondary outcome was students’ satisfaction with FC. Two researchers independently extracted the data, and disagreements were resolved by consensus with a third researcher.

### 2.4 Risk of bias assessment and certainty of evidence measurement

The evaluation of bias risk in each RCT was conducted by two independent reviewers using the Cochrane Risk of Bias Assessment Tool (version 2), and the disagreement was resolved by the third researcher (18). For cohort study and quasi-experimental study, we used the “Risk of Bias” tool from the Cochrane Effective Practice and Organization of Care Group (EPOC) (19), which used generation of randomization sequence, concealment of treatment allocation, similarity of baseline outcome measurement, similarity of baseline characteristics, incomplete outcome reporting, adequacy of blinding to intervention assignment, protection against contamination, selective outcome reporting, and other risks of bias. According to the description of each study, the assessment of each area is marked as “low risk,” “high risk,” or “unclear risk.” Any differences shall be resolved through discussion until consensus is reached. Additionally, the Grading of Recommendations, Assessment, Development, and Evaluations (GRADE) system was applied to measure the certainty of evidence (CoE) (GRADEpro GDT software, https://www.gradepro.org) (20). For each outcome, the certainty of evidence was assessed separately for randomized and non-randomized studies.

### 2.5 Subgroup analyses

To identify possible sources of heterogeneity and the variability in effects of FC versus TL across contexts, subgroup analyses were performed. We stratified pooled effects by: (a) study design (RCT, Cohort Study, Quasi-Experimental Study); (b) study direction (prospective and retrospective); (c) continent (America, Asia, Europe); (d) curriculum type including surgery clerkship, basic surgical skills training (e.g., fundamental operation skill and laparoscopic suturing), surgical subspecialty curriculum (e.g., urology, neurosurgery); (e) exam type is categorized as standardized exam and custom-designed exam (standardized exams including National Board of Medical Examiners (NBME) and Objective Structured Clinical Examinations (OSCE), are conducted under predefined, uniform frameworks governing administration and scoring; custom-designed exams are defined as investigator-developed, course-specific, non-standardized assessments).

### 2.6 Statistical analysis

Statistical analysis was conducted using Review Manager (version 5.3) and Stata (version 18.0) software. Continuous variables were analyzed using either the standard mean differences (SMDs) or mean differences (MDs) with 95% confidence intervals (CIs). The chi-square test and *I*^2^ statistic were employed to assess statistical heterogeneity. The fixed-effects model was applied when heterogeneity was low (*I*^2^ < 50%), and the random-effects model was adopted if the heterogeneity was high in the pooled studies (*I*^2^ > 50%). For studies that reported only the median and range, the sample mean and standard deviation were estimated using the method proposed by Wan et al. (21). Publication bias was evaluated using funnel plots, along with Egger’s and Begg’s tests. A sensitivity analysis was conducted to test the robustness of the pooled effects by sequentially removing each study. Statistical significance was defined as *p* < 0.05.

## 3 Results

### 3.1 Search results and study selection

**Figure 1:**
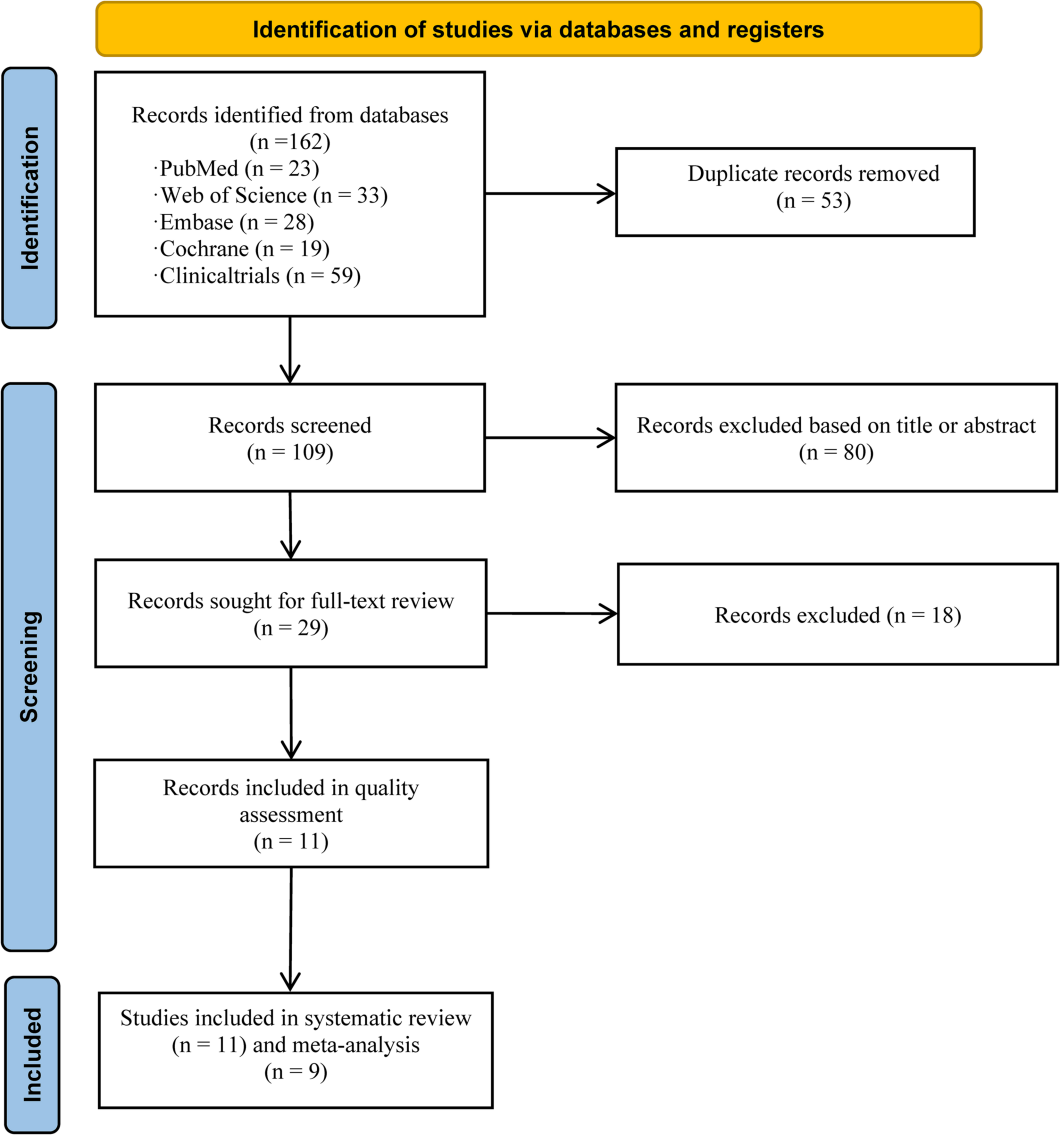
PRISMA flow chart of study retrieval.

Initially, a total of 162 studies were obtained from 5 databases using the predefined search strategy. After the duplication, 109 studies remained and were screened. By reviewing the title and abstract, 80 studies were removed. A total of 29 studies were enrolled in full-text screening, and 18 of them were excluded. Finally, 11 studies were included for systematic review (6, 14, 15, 22–29) and nine studies for meta-analysis (6, 14, 15, 23–26, 28, 29). The study selection was performed following the PRISMA flow diagram (Figure 1).

### 3.2 Characteristics of the studies

Among all 11 included studies, five studies were RCT (22, 24–26, 28), five studies were cohort study (6, 14, 23, 27, 29), and another was quasi-experimental study (15). 10 studies were prospective (6, 14, 15, 22, 24–29), and only one study was retrospective (23). Five studies were conducted in Asia (22, 24, 26, 28, 29), four were conducted in America (6, 14, 23, 27), and the other two were conducted in Europe (15, 25). The sample size ranged from 17 to 323.

Every study provided a clear identification of FC and implemented TL in control group. Regarding students’ academic performance outcomes, 11 studies reported the total score (6, 14, 15, 22–29), eight the theoretical score (15, 22, 23, 25–29), and four the operational score (24, 27–29). Moreover, 10 studies examined students’ satisfaction with FC (6, 14, 15, 22, 24–29). Additional details are provided in Table 1.

**Table 1:** Characteristics of included studies in the meta-analysis.

Study	Country	Study design	Subjects	Participant description	Sample size (intervention/control)	Intervention	Control	Study duration	Study outcomes
Zhou et al. (29)	China	Prospective single-center cohort study	Fundamental operations in surgery	8-year program students (juniors) majored in clinical medicine	64/58	SPOC+FC	TL	One academic year	①②③④
Wang et al. (28)	China	Single-center RCT	Orthopedic surgery	Undergraduate 4th-year students	69/69	FC	TL	NR	①②③④
Tassavor et al. (27)	USA	Prospective single-center cohort study	Dermatologic surgery	Residents	17 in total	FC	TL	1 month	①②③④
Liebert et al. (14)	USA	Prospective single-center cohort study	Surgery core clerkship	3rd-year medical students	89/92	FC	TL	One academic year	①④
Lewis et al. (6)	USA	Prospective Single-center cohort study	surgery clerkship	3rd-year medical students	98/102	FC	TL	One academic year	①④
Gutiérrez-González et al. (15)	Spain	Quasi-experimental study	Neurosurgery	4th-year undergraduate medical students	69/187	FC	TL	One academic year	①②④
Elledge et al. (25)	UK	Single-center RCT	Maxillofacial surgery	NR	10/14	FC	TL	NR	①②④
Barrett et al. (23)	USA	Retrospective single-center cohort	Surgery core clerkship	3rd-year medical students	166/157	FC	TL	8 weeks	①②
Ng et al. (26)	China	Single-center RCT	Urological surgery	4th to 6th-year medical students	23/22	FC	TL	3 weeks	①②④
Alharbi et al. (22)	Saudi Arab	Single-center RCT	Orthodontics & orthognathic surgery	4th-year undergraduate dental students	16/16	FC	TL	3 weeks	①②④
Chiu et al. (24)	China	Single-center RCT	Laparoscopic suturing skills	6th-year medical students	30/29	FC	TL	1 hour session	①③④

① Total scores; ② Theoretical scores; ③ Operational scores; ④ Students’ satisfaction with flipped classroom. FC, flipped classroom. TL, traditional lecture. NR, not reported.

### 3.3 Study quality assessment

**Figure 2:**
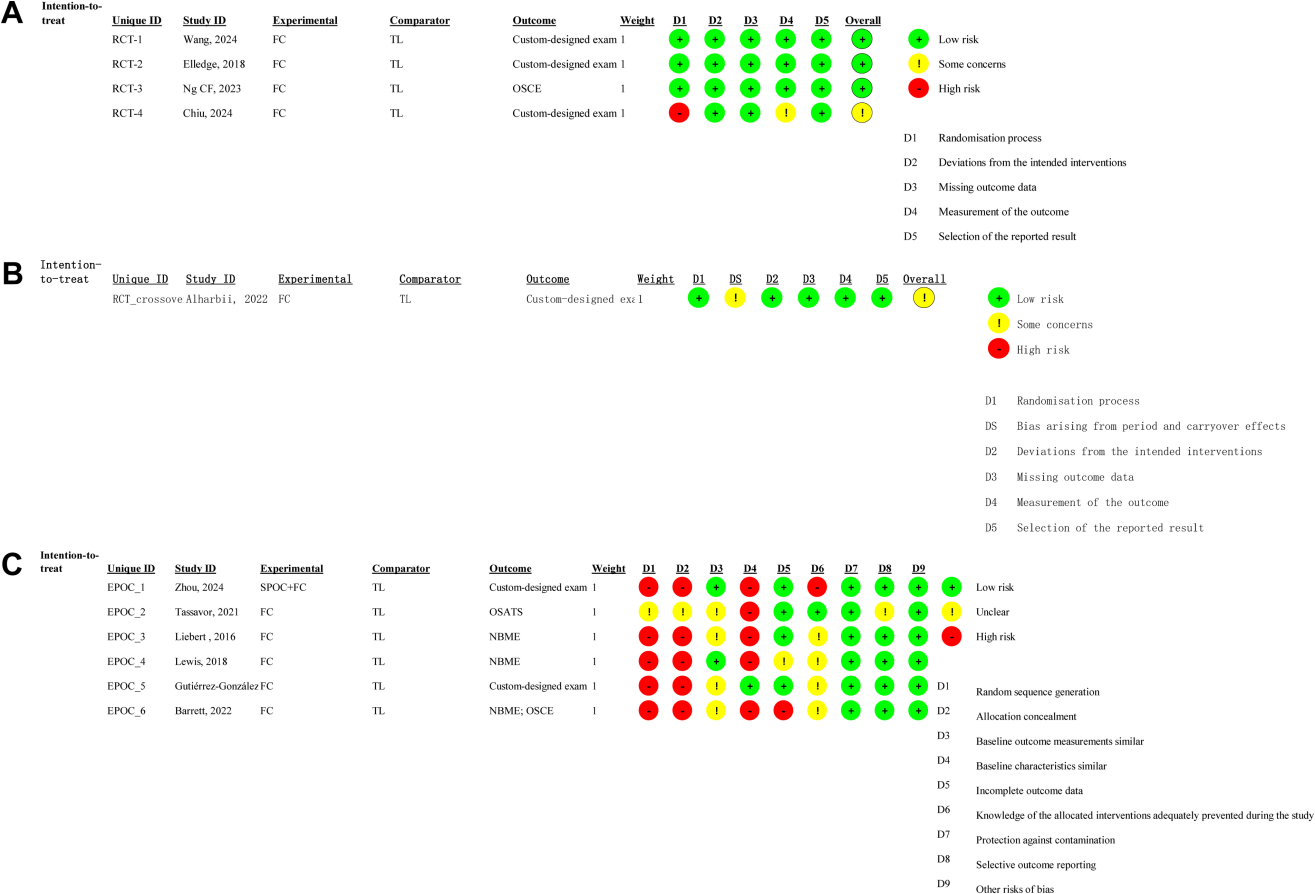
Quality assessment of the included studies by RoB2.0 tool **(A)** parallel-group randomized controlled trial, **(B)** crossover trial and EPOC tool **(C)** cohort study and quasi-experimental study.

Among the RCTs, three studies explicitly reported both randomization and the method of sequence generation, and were therefore deemed at low risk of bias arising from the randomization process. One trial employed a registered order for allocation and was rated as high risk. All four parallel-group RCTs provided complete primary outcome data, resulting in a low risk of attrition bias. In the single crossover trial, the randomization process was considered low risk; however, concerns were raised regarding period and carryover effects because no washout period was specified. For cohort studies and quasi-experimental study, bias from random sequence generation and allocation concealment were rated as high in five studies, and bias from incomplete outcome data were assessed as low in four studies. The risk of bias assessment is summarized and shown in Figure 2.

### 3.4 Effects of flipped classroom on student learning outcomes in surgical education

#### 3.4.1 Total scores

**Figure 3:**
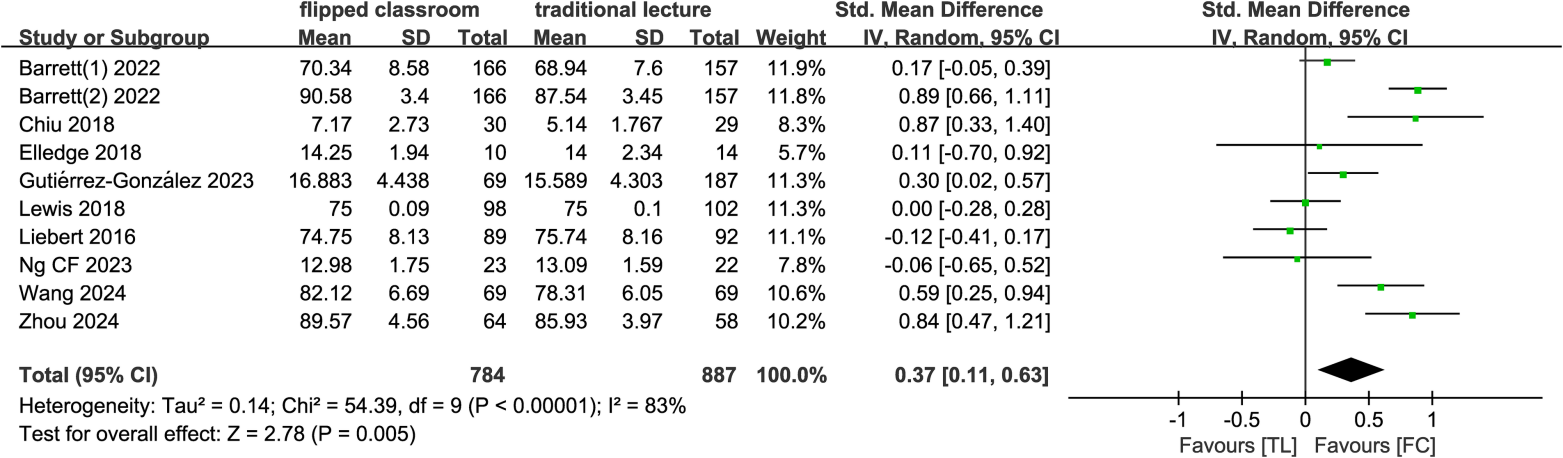
Forest plot for the effects of FC on total scores compared with TL.

**Figure 4:**
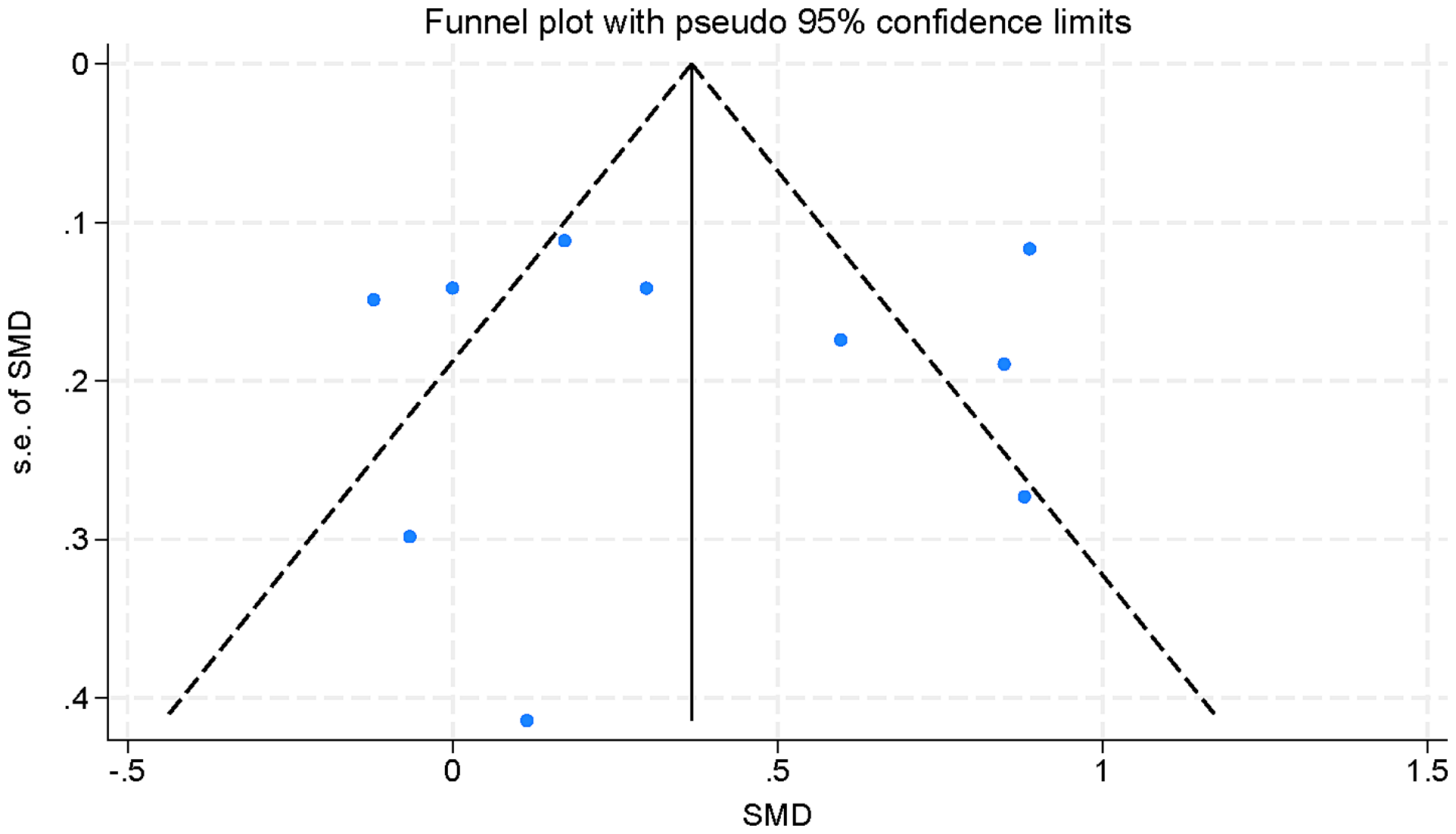
Publication bias of total scores assessed by funnel plot.

Nine studies involving ten cohorts and a total of 1,348 participants (618 in the FC group and 730 in the TL group) reported total scores. The pooled data indicated a significant benefit of the FC approach over the TL method in surgical education as measured by increased total scores (SMD = 0.37, 95% CI: 0.11–0.63, *p* = 0.005, *I^2^* = 83%) (Figure 3). Two studies could not be included in the pooled analysis. A statistically significant difference in Objective Structured Assessment of Technical Skills scores favoring the FC group over the TL group was reported by Tassavor et al. (*p* = 0.0287), but only the total sample size was reported without group-specific data. Alharbi et al. found no significant differences between the FC group and the TL group in both crossover periods (*p* > 0.05), but this study provided insufficient outcome data for meta-analysis. The funnel plot suggested no significant publication bias. (Egger’s test: *p* = 0.997, Begg’s test: *p* = 0.858) (Figure 4). The GRADE assessment indicated high CoE in randomized studies and very low CoE in non-randomized studies (Supplementary Figure 2A).

#### 3.4.2 Theoretical scores

**Figure 5:**
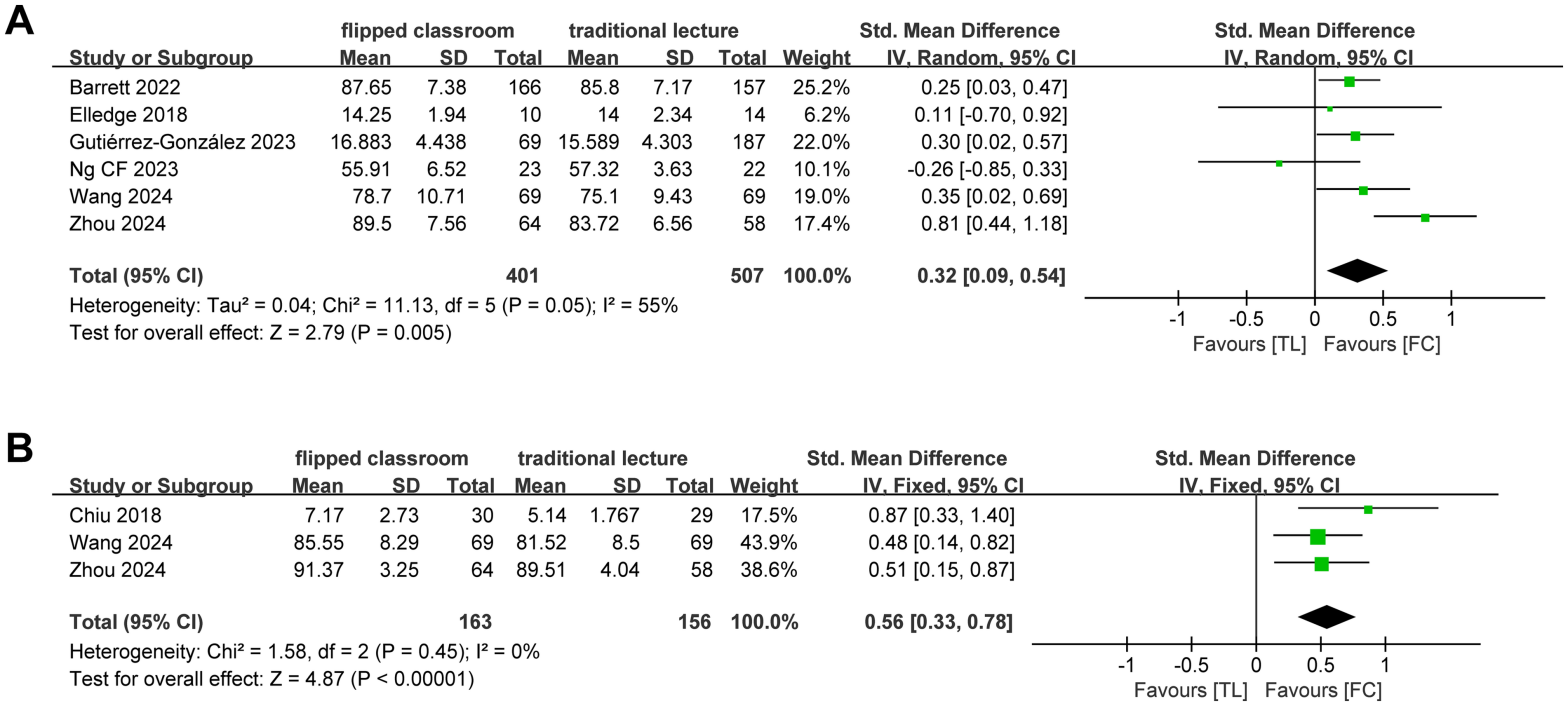
Forest plot for the effects of FC on theoretical scores **(A)** and operational scores **(B)** compared with TL.

Six studies involving 908 participants (401 in the FC group and 507 in the TL group) reported the theoretical scores. The pooled data suggested improved theoretical learning outcomes with the FC approach compared to the TL method (SMD = 0.32, 95% CI: 0.09–0.54, *p* = 0.005, *I*^2^ = 55%) (Figure 5A). The funnel plot did not show evidence of significant publication bias (Egger’s test: *p* = 0.744, Begg’s test: *p* = 0.851) (Supplementary Figure 1A). The GRADE assessment indicated moderate CoE in randomized studies and very low CoE in non-randomized studies (Supplementary Figure 2B).

#### 3.4.3 Operational scores

Three studies involving 319 participants (163 in the FC group and 156 in the TL group) reported the operational scores. The pooled data showed improved operational learning outcomes with the FC approach when compared with the TL method (SMD = 0.56, 95% CI: 0.33–0.78, *p* < 0.00001, *I*^2^ = 0%) (Figure 5B). Egger’s test showed significant evidence of publication bias (*p* = 0.002), while Begg’s test did not reveal significant asymmetry (*p* = 0.296) (Supplementary Figure 1B). The GRADE assessment indicated high CoE in randomized studies and very low CoE in non-randomized studies (Supplementary Figure 2C).

#### 3.4.4 Subgroup analysis

Subgroup analyses indicated that study design, study direction, continent, curriculum type, and exam type may contribute to heterogeneity. In terms of study design, cohort study showed marked heterogeneity, whereas RCT was relatively consistent. For study direction, heterogeneity was considerable in prospective study and high in retrospective study. Across continents, substantial heterogeneity was observed in America compared with Asia and Europe. Regarding curriculum, surgery clerkship showed considerable heterogeneity, while basic surgical skills training remained homogeneous. Finally, heterogeneity was moderate in custom-designed exam but pronounced in OSCE. The results also showed that FC has a favorable effect over TL across subgroups. Detailed findings are presented in Table 2.

**Table 2:** Subgroup analyses of the effects of flipped classroom versus traditional lecture across study design, study direction, continent, curriculum, and exam.

Subgroup	*n*	Effects		Heterogeneity	
		SMD (95% CI)	*p* value	*I*^2^, %	*p* value
Study design					
RCT	4	0.43 [0.03, 0.83]	0.03	54%	0.09
Cohort study	5	0.35 [−0.06, 0.76]	0.09	91%	< 0.00001
Quasi-experimental study	1	0.30 [0.02, 0.57]	0.043		
Study direction					
Prospective	8	0.32 [0.04, 0.60]	0.03	76%	0.0001
Retrospective	2	0.53 [−0.17, 1.23]	0.14	95%	< 0.00001
Continent					
America	4	0.24 [−0.21, 0.69]	0.3	92%	< 0.00001
Asia	4	0.60 [0.25, 0.95]	0.0008	60%	0.06
Europe	2	0.28 [0.02, 0.54]	0.04	0%	0.67
Curriculum					
Surgery clerkship	4	0.24 [−0.21, 0.69]	0.3	92%	< 0.00001
Basic surgical skills training	2	0.85 [0.55, 1.16]	< 0.00001	0%	0.94
Surgical subspecialty curriculum	4	0.34 [0.15, 0.54]	0.0006	31%	0.22
Exam					
NBME	3	0.05 [−0.10, 0.20]	0.53	24%	0.27
OSCE	2	0.45 [−0.48, 1.38]	0.34	89%	0.003
Custom-designed exam	5	0.57 [0.30, 0.83]	< 0.0001	51%	0.09

SMD, standard mean difference; CI, confidence interval; RCT, randomized controlled trial; NBME, National Board of Medical Examiners; OSCE, Objective Structured Clinical Examinations.

#### 3.4.5 Sensitivity analysis

Leave-one-out sensitivity analysis showed that the overall effects remained stable (pooled SMDs from 0.29 to 0.43). The heterogeneity remained robust with *I^2^* ranging from 73 to 85%. The smallest changes in heterogeneity occurred when Lewis et al. or Zhou et al. was removed, with the pooled SMDs shifting from 0.37 to 0.41 and 0.31, respectively. Only one study (Barrett et al., OSCE cohort) contributing notably to heterogeneity of sensitivity. Excluding this cohort reduced *I^2^* from 83 to 73% and changed the pooled SMDs from 0.37 to 0.29. See the details in Supplementary Table 2.

#### 3.4.6 Students’ satisfaction with FC

In this systematic review and meta-analysis, numerous studies reported that students in FC groups were satisfied with the teaching method and were interested in FC courses. Several studies employing 5-point Likert scales showed that most students rated FC positively with high scores on satisfaction-related items (14, 15, 24). Zhou et al. (29) used a 6-item questionnaire, and students’ responses reflected high satisfaction with FC, expressing their interest and enthusiasm in FC courses. Moreover, two studies compared FC group with TL group regarding satisfaction and perceptions, indicating that students in FC group had more positive course experiences (22, 28). In addition, three studies reported increased confidence among students in FC group (25–27).

## 4 Discussion

This systematic review and meta-analysis investigated the effects of FC on student learning outcomes in surgical education. Our key findings suggest that the FC approach presented more positive effects over the TL method in improving students’ academic performance including the total scores, theoretical scores, and operational scores. We also found that FC is related to enhanced satisfaction of students. These findings indicate that FC is a potential approach in surgical education.

The greater effectiveness of FC compared with TL may be explained by several factors. In the FC model, students typically access online lectures and other study materials before class, enabling them to engage in personalized learning at their own pace (8). Such self-directed study allows learners to manage their study time flexibly and to focus on targeted content tailored to their needs, thereby enhancing learning efficiency (12, 30). During class sessions, instructors act as facilitators, guiding students through various student-centered learning activities such as group discussions and problem-solving exercises (31). This instructional role enables teachers to provide timely clarification and feedback, which helps to guide and promote students’ learning (32). Moreover, the FC approach encourages students to apply what they have learned through problem-solving and collaborative learning, thus deepening their understanding and mastery of the subject matter (7, 33). Additionally, compared with passive learning, students in flipped classrooms actively interact with peers and instructors. This active engagement enhances their attention, improves learning efficiency, and results in deeper learning. Our findings are consistent with other systematic reviews in fields such as radiology, nursing, and pharmacy (10, 34, 35), underscoring the broad applicability of FC model in medical education.

Surgical education encompasses not only theoretical knowledge but also practical and operational skills. TL-based teaching mainly focuses on theoretical content and is less effective in demonstrating complex procedural skills (36). By contrast, FC model provides students with access to visual and repeatable learning resources, such as online videos and simulation materials, which facilitate their mastery of surgical skills and enhance confidence (27, 37). Furthermore, FC reserves class time for skills practice, allowing instructors to provide targeted guidance and feedback. As a result, FC enhances the proficiency and quality of students’ surgical skills, which may explain our finding of significantly improved operational scores. Moreover, surgery requires a broad body of multidisciplinary knowledge, such as anatomy, physiology, and pathology, which makes the subject extensive and challenging. TL-based teaching can easily lead to cognitive overload, reducing learning efficiency (11). FC addresses this challenge by enabling self-directed learning before class and guided application of knowledge during class, thereby supporting information processing and knowledge retention (30). In addition, compared with basic science courses, surgery is more clinically oriented, requiring the development of clinical reasoning. FC strengthens knowledge application and critical thinking through case-based discussions and other active learning activities, thus improving clinical reasoning skills (38, 39).

Notably, our study identified that FC is associated with high student satisfaction and interest, a finding similar to previous research (40, 41). We believe this may be related to the increased classroom interaction and engagement. In the FC model, both peer-to-peer and student–instructor interactions are enhanced (32). As a result, students are able to receive timely responses and recognition during the learning process, which in turn enhances their satisfaction. Furthermore, when students take an active role and have greater control over their learning activities, they develop a sense of ownership that enhances their interest and confidence, resulting in a more positive learning experience (8). Higher satisfaction and positive learning experiences are likely to have a positive effect on students’ learning.

The subgroup analyses provide valuable insights into both the sources of heterogeneity and the variability in effects of FC across contexts. First, study design appeared to contribute to heterogeneity, which may be attributable to the lack of randomization in cohort studies (42). Second, geographic differences may also account for heterogeneity. Studies conducted in Asia demonstrated relatively larger and more consistent effects, whereas those from America reported smaller and less consistent effects, suggesting that FC may confer greater advantages in specific regional or educational contexts. Third, both curriculum type and exam format appeared to influence the observed effects and heterogeneity. Basic surgical skills training demonstrated substantial and consistent effects, which may be attributed to the emphasis on practice in FC (43, 44). However, surgery clerkship exhibited high heterogeneity. Given its focus on both surgical knowledge and clinical practice, the effects of FC depend more on the teaching faculty. Differences in faculty experience and teaching styles may therefore have a stronger influence and contribute to this heterogeneity. In addition, surgery clerkship usually involves extensive clinical case discussions that cover a wide range of subjects and topics, which could result in notable variability in learning outcomes. Compared with standardized tests, custom-designed exams yielded larger effects with moderate heterogeneity, indicating that tailored assessments may be better suited to FC teaching. In contrast, OSCE showed high heterogeneity, which may be due to the subjective nature of scoring by standardized patients and variability in their performance (45). Nevertheless, unexplained sources of heterogeneity remain and should be explored in subsequent studies. These findings therefore suggest that the pooled estimates should be interpreted with caution when generalizing the results to other medical education settings.

Although our study demonstrated the significant effectiveness of FC in surgical education, its practical application still requires further refinement. In the future, educators should consider providing students with innovative pre-class materials and resources that are specifically tailored to the demands of surgical education, such as virtual reality and simulation-based modules (46, 47). During in-class sessions, emphasis should be placed on skills practice and knowledge application to align with the hands-on and decision-making nature of surgical practice (48). Furthermore, assessment strategies should be diversified, as FC not only facilitates students’ mastery of theoretical knowledge but also enhances their clinical reasoning and critical thinking skills (11, 38). Evaluations of teachers’ satisfaction should also be included in future studies as teachers directly influence the implementation quality and sustainability of FC. Perspectives from affiliated teaching hospitals or clinical employers should also be considered to assess whether FC improves students’ readiness for real clinical practice. As FC becomes more widely used in medical education, many educators have begun conducting cohort studies, quasi-experimental studies, and RCTs, to explore the impact of FC. These studies generally suggest that FC is effective. Looking ahead, more rigorously designed, multi-center, and even multi-disciplinary studies will be important to provide clearer evidence on the effectiveness of FC. In surgical education, current applications of FC are largely limited to surgery clerkships and certain specific disciplines such as orthopedics and urology. Future RCTs are therefore needed to cover a broader range of surgical courses, including surgical theory, surgical skills training, and various surgical disciplines. Moreover, future studies may need to use RCTs to validate the effectiveness of FC across different outcomes involve theoretical exams, surgical operations, clinical performance, or satisfaction reported by both students and teachers.

This study has several notable strengths. To our knowledge, this is the first systematic review and meta-analysis to explore the effectiveness of FC in surgical education, addressing an important gap in medical education. We included course total scores, theoretical scores, and operational scores in the meta-analysis, which provided a more comprehensive evaluation of the effects of FC across both knowledge acquisition and skills performance. In addition, we applied a previously validated algorithm to estimate the mean and standard deviation of one study that did not report these values, increasing the number of studies and the overall sample size in the meta-analysis. Finally, the CoE was assessed using the GRADE approach, which reinforced the reliability of this meta-analysis and provided additional information on FC in surgical education.

However, several limitations should be acknowledged. First, due to the inherent characteristics of educational research, many included studies were at high risk of bias in randomization and allocation concealment. Second, high heterogeneity was observed in the pooled total scores. Although subgroup analyses partially explained the heterogeneity, it may also have arisen from other factors such as individual learning styles or differences in study interventions. Therefore, high heterogeneity should be considered when interpreting and generalizing our findings, as the impact of FC may vary across different contexts and methodologies. Third, the number of included RCTs was limited, which may lead to low evidence quality regarding the effectiveness of FC. In addition, the outcomes of students’ feedback could not be included in the meta-analysis due to the diversity in the assessment tools, scoring scales, and reporting formats across the included studies. This decreased the evidence robustness of the effect of FC on students’ satisfaction and highlighted the urgent need for future research to adopt more standardized, validated instruments and consistent reporting methods when evaluating students’ satisfaction. Furthermore, most included studies assessed only short-term learning outcomes such as course scores and students’ satisfaction, resulting in a lack of long-term outcomes. Additionally, the GRADE assessment showed non-randomized studies have very low CoE and conclusions are mainly supported by RCTs.

## 5 Conclusion

This systematic review and meta-analysis suggests that the FC approach may be more effective than the TL method for improving students’ academic performance and satisfaction in surgical education. However, non-randomized studies have very low GRADE certainty, and our conclusions are therefore primarily supported by the available RCTs. FC may be regarded as a promising instructional tool for conducting surgical education. More high-quality RCTs are expected to confirm these findings in the future.

## Data Availability

The original contributions presented in the study are included in the article/Supplementary material, further inquiries can be directed to the corresponding authors.
