# Supplementary material for: Application of flipped classroom in surgical education: a systematic review and meta-analysis
Source: Front Med (Lausanne). 2026 May 28;13:1841948. doi: 10.3389/fmed.2026.1841948 (PMC13253294; doi:10.3389/fmed.2026.1841948)
Supplement: Supplementary file 2 [file Table_2.docx]

Supplementary Table 1. Detailed search strategy.

Supplementary Table 2. Leave-one-out sensitivity analysis of the pooled effect size and heterogeneity.

**Supplementary Table 1**. Detailed search strategy.

| Web of Science | TI=("flipped classroom" OR "flipped class" OR "flipping the classroom" OR "flipped learning" OR "flipped instruction" OR "inverted classroom" OR "reverse classroom" OR "inverse classroom" OR "flip classroom" OR "classroom flip" OR "education flipped" OR "classroom inverted" OR "flip learning" OR "flip education" OR "flip instruction" OR "flip class" OR "flipped learning" OR "flipped education" OR "flipped class" OR "flipping classroom" OR "flipping education" OR "flipping instruction" OR "flipping class")  AND  TI=("General Surgery" OR "Cardiothoracic Surgery" OR "Thoracic Surgery" OR "Cardiac Surgery" OR "Colorectal Surgery" OR "Endocrine Surgery" OR "Gastrointestinal Surgery" OR "Hand Surgery" OR "Hepatobiliary Surgery" OR "Maxillofacial Surgery" OR "Oral and Maxillofacial Surgery" OR Neurosurgery OR "Obstetric Surgery" OR "Surgical Oncology" OR "Oncological Surgery" OR "Ophthalmic Surgery" OR "Eye Surgery" OR "Orthopaedic Surgery" OR "Orthopedic Surgery" OR "ENT Surgery" OR Otorhinolaryngology OR Otolaryngology OR "Paediatric Surgery" OR "Pediatric Surgery" OR "Plastic Surgery" OR "Podiatric Surgery" OR "Foot and Ankle Surgery" OR "Spinal Surgery" OR "Trauma and Orthopaedics" OR "Trauma and Orthopedics" OR "Urological Surgery" OR "Urologic Surgery" OR "Vascular Surgery" OR "Burn Surgery" OR "Field Surgery" OR Urology OR Orthopedics OR Orthopaedics OR Surgery OR Surgical) |
| --- | --- |
| PubMed | ("flipped classroom"[Title] OR "flipped class"[Title] OR "flipping the classroom"[Title] OR "flipped learning"[Title] OR "flipped instruction"[Title] OR "inverted classroom"[Title] OR "reverse classroom"[Title] OR "inverse classroom"[Title] OR "flip classroom"[Title] OR "classroom flip"[Title] OR "education flipped"[Title] OR "classroom inverted"[Title] OR "flip learning"[Title] OR "flip education"[Title] OR "flip instruction"[Title] OR "flip class"[Title] OR "flipped learning"[Title] OR "flipped education"[Title] OR "flipped class"[Title] OR "flipping classroom"[Title] OR "flipping education"[Title] OR "flipping instruction"[Title] OR "flipping class"[Title])  AND  ("General Surgery"[Title] OR "Cardiothoracic Surgery"[Title] OR "Thoracic Surgery"[Title] OR "Cardiac Surgery"[Title] OR "Colorectal Surgery"[Title] OR "Endocrine Surgery"[Title] OR "Gastrointestinal Surgery"[Title] OR "Hand Surgery"[Title] OR "Hepatobiliary Surgery"[Title] OR "Maxillofacial Surgery"[Title] OR "Oral and Maxillofacial Surgery"[Title] OR Neurosurgery[Title] OR "Obstetric Surgery"[Title] OR "Surgical Oncology"[Title] OR "Oncological Surgery"[Title] OR "Ophthalmic Surgery"[Title] OR "Eye Surgery"[Title] OR "Orthopaedic Surgery"[Title] OR "Orthopedic Surgery"[Title] OR "ENT Surgery"[Title] OR Otorhinolaryngology[Title] OR Otolaryngology[Title] OR "Paediatric Surgery"[Title] OR "Pediatric Surgery"[Title] OR "Plastic Surgery"[Title] OR "Podiatric Surgery"[Title] OR "Foot and Ankle Surgery"[Title] OR "Spinal Surgery"[Title] OR "Trauma and Orthopaedics"[Title] OR "Trauma and Orthopedics"[Title] OR "Urological Surgery"[Title] OR "Urologic Surgery"[Title] OR "Vascular Surgery"[Title] OR "Burn Surgery"[Title] OR "Field Surgery"[Title] OR Urology[Title] OR Orthopedics[Title] OR Orthopaedics[Title] OR Surgery[Title] OR Surgical[Title]) |
| Embase | ('flipped classroom':ti OR 'flipped class':ti OR 'flipping the classroom':ti OR 'flipped learning':ti OR 'flipped instruction':ti OR 'inverted classroom':ti OR 'reverse classroom':ti OR 'inverse classroom':ti OR 'flip classroom':ti OR 'classroom flip':ti OR 'education flipped':ti OR 'classroom inverted':ti OR 'flip learning':ti OR 'flip education':ti OR 'flip instruction':ti OR 'flip class':ti OR 'flipped learning':ti OR 'flipped education':ti OR 'flipped class':ti OR 'flipping classroom':ti OR 'flipping education':ti OR 'flipping instruction':ti OR 'flipping class':ti)  AND  ('General Surgery':ti OR 'Cardiothoracic Surgery':ti OR 'Thoracic Surgery':ti OR 'Cardiac Surgery':ti OR 'Colorectal Surgery':ti OR 'Endocrine Surgery':ti OR 'Gastrointestinal Surgery':ti OR 'Hand Surgery':ti OR 'Hepatobiliary Surgery':ti OR 'Maxillofacial Surgery':ti OR 'Oral and Maxillofacial Surgery':ti OR Neurosurgery:ti OR 'Obstetric Surgery':ti OR 'Surgical Oncology':ti OR 'Oncological Surgery':ti OR 'Ophthalmic Surgery':ti OR 'Eye Surgery':ti OR 'Orthopaedic Surgery':ti OR 'Orthopedic Surgery':ti OR 'ENT Surgery':ti OR Otorhinolaryngology:ti OR Otolaryngology:ti OR 'Paediatric Surgery':ti OR 'Pediatric Surgery':ti OR 'Plastic Surgery':ti OR 'Podiatric Surgery':ti OR 'Foot and Ankle Surgery':ti OR 'Spinal Surgery':ti OR 'Trauma and Orthopaedics':ti OR 'Trauma and Orthopedics':ti OR 'Urological Surgery':ti OR 'Urologic Surgery':ti OR 'Vascular Surgery':ti OR 'Burn Surgery':ti OR 'Field Surgery':ti OR Urology:ti OR Orthopedics:ti OR Orthopaedics:ti OR Surgery:ti OR Surgical:ti) |
| Cochrane Library | #1 ("flipped classroom":ti,ab,kw OR "flipped class":ti,ab,kw OR "flipping the classroom":ti,ab,kw OR "flipped learning":ti,ab,kw OR "flipped instruction":ti,ab,kw OR "inverted classroom":ti,ab,kw OR "reverse classroom":ti,ab,kw OR "inverse classroom":ti,ab,kw OR "flip classroom":ti,ab,kw OR "classroom flip":ti,ab,kw OR "education flipped":ti,ab,kw OR "classroom inverted":ti,ab,kw OR "flip learning":ti,ab,kw OR "flip education":ti,ab,kw OR "flip instruction":ti,ab,kw OR "flip class":ti,ab,kw OR "flipped learning":ti,ab,kw OR "flipped education":ti,ab,kw OR "flipped class":ti,ab,kw OR "flipping classroom":ti,ab,kw OR "flipping education":ti,ab,kw OR "flipping instruction":ti,ab,kw OR "flipping class":ti,ab,kw)  #2 ("General Surgery":ti,ab,kw OR "Cardiothoracic Surgery":ti,ab,kw OR "Thoracic Surgery":ti,ab,kw OR "Cardiac Surgery":ti,ab,kw OR "Colorectal Surgery":ti,ab,kw OR "Endocrine Surgery":ti,ab,kw OR "Gastrointestinal Surgery":ti,ab,kw OR "Hand Surgery":ti,ab,kw OR "Hepatobiliary Surgery":ti,ab,kw OR "Maxillofacial Surgery":ti,ab,kw OR "Oral and Maxillofacial Surgery":ti,ab,kw OR Neurosurgery:ti,ab,kw OR "Obstetric Surgery":ti,ab,kw OR "Surgical Oncology":ti,ab,kw OR "Oncological Surgery":ti,ab,kw OR "Ophthalmic Surgery":ti,ab,kw OR "Eye Surgery":ti,ab,kw OR "Orthopaedic Surgery":ti,ab,kw OR "Orthopedic Surgery":ti,ab,kw OR "ENT Surgery":ti,ab,kw OR Otorhinolaryngology:ti,ab,kw OR Otolaryngology:ti,ab,kw OR "Paediatric Surgery":ti,ab,kw OR "Pediatric Surgery":ti,ab,kw OR "Plastic Surgery":ti,ab,kw OR "Podiatric Surgery":ti,ab,kw OR "Foot and Ankle Surgery":ti,ab,kw OR "Spinal Surgery":ti,ab,kw OR "Trauma and Orthopaedics":ti,ab,kw OR "Trauma and Orthopedics":ti,ab,kw OR "Urological Surgery":ti,ab,kw OR "Urologic Surgery":ti,ab,kw OR "Vascular Surgery":ti,ab,kw OR "Burn Surgery":ti,ab,kw OR "Field Surgery":ti,ab,kw OR Urology:ti,ab,kw OR Orthopedics:ti,ab,kw OR Orthopaedics:ti,ab,kw OR Surgery:ti,ab,kw OR Surgical:ti,ab,kw)  #3 #1 AND #2 |
| ClinicalTrials.gov | #1 flipped classroom  #2 inverse classroom  #3 inverted classroom  #4 reverse classroom |

**Supplementary Table 2**. Leave-one-out sensitivity analysis of the pooled effect size and heterogeneity.

|  | **Effects** |  | **Heterogeneity** |  |  |
| --- | --- | --- | --- | --- | --- |
|  | **SMD (95%CI)** | ***P* value** | ***I^2^,* %** | ***P* value** | **Tau²** |
| Overall | 0.37[0.11, 0.63] | 0.005 | 83% | < 0.00001 | 0.14 |
| Barrett(1) | 0.39[0.10, 0.69] | 0.01 | 84% | < 0.00001 | 0.16 |
| Barrett(2) | 0.29[0.07, 0.52] | 0.01 | 73% | 0.0002 | 0.08 |
| Chiu | 0.32[0.05, 0.59] | 0.02 | 84% | < 0.00001 | 0.13 |
| Elledge | 0.38[0.11, 0.65] | 0.006 | 85% | < 0.00001 | 0.14 |
| Gutiérrez-González | 0.38[0.08, 0.67] | 0.01 | 85% | < 0.00001 | 0.16 |
| Lewis | 0.41[0.14, 0.69] | 0.003 | 83% | < 0.00001 | 0.14 |
| Liebert | 0.43[0.17, 0.69] | 0.001 | 81% | < 0.00001 | 0.12 |
| Ng CF | 0.40[0.13, 0.68] | 0.004 | 85% | < 0.00001 | 0.14 |
| Wang | 0.34[0.06, 0.62] | 0.02 | 85% | < 0.00001 | 0.15 |
| Zhou | 0.31[0.04, 0.58] | 0.02 | 83% | < 0.00001 | 0.13 |
