# Supplementary material for: Application of flipped classroom in surgical education: a systematic review and meta-analysis
Source: Front Med (Lausanne). 2026 May 28;13:1841948. doi: 10.3389/fmed.2026.1841948 (PMC13253294; doi:10.3389/fmed.2026.1841948)
Supplement: Supplementary file 3 [file Image_1.pdf]

Supplementary Figure 1. Funnel plots for publication bias of theoretical scores (A) and operational scores (B).

Supplementary Figure 2. Certainty of evidence for total scores (A), theoretical scores (B), and operational scores (C) measured by the GRADE system.

A

B

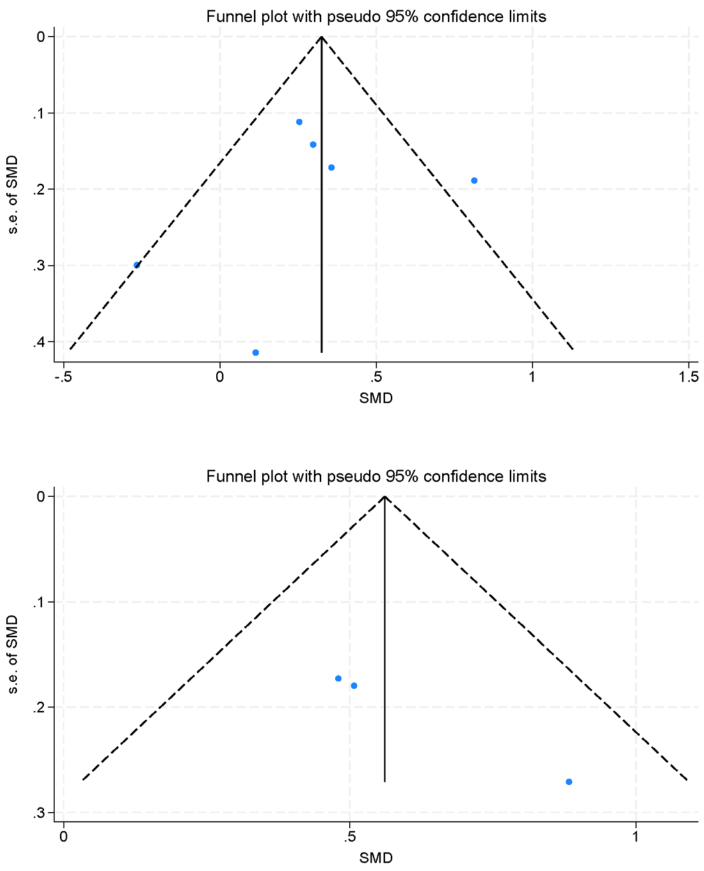

Supplementary Figure 1. Funnel plots for publication bias of theoretical scores (A) and operational scores (B).

A

Author(s):
Question: FC compared to TL for surgical education
Setting:
Bibliography:

Certainty assessment							№ of patients		Effect		Certainty	Importance
№ of studies	Study design	Risk of bias	Inconsistency	Indirectness	Imprecision	Other considerations	FC	TL	Relative (95% CI)	Absolute (95% CI)		
**Total scores_randomised studies**												
4	randomised trials	not serious	not serious	not serious	not serious	none	132	134	-	SMD **0.43 SD higher** (0.03 higher to 0.83 higher)	⨁⨁⨁⨁ High	CRITICAL
**Total scores_non-randomised studies**												
6	non-randomised studies	serious	serious	not serious	not serious	none	652	753	-	SMD **0.34 SD higher** (0.01 higher to 0.68 higher)	⨁◯◯◯ Very low	CRITICAL

CI: confidence interval; **SMD:** standardised mean difference

B

Author(s):
Question: FC compared to TL for surgical education
Setting:
Bibliography:

Certainty assessment							№ of patients		Effect		Certainty	Importance
№ of studies	Study design	Risk of bias	Inconsistency	Indirectness	Imprecision	Other considerations	FC	TL	Relative (95% CI)	Absolute (95% CI)		
**Theoretical scores_randomised studies**												
3	randomised trials	not serious	not serious	not serious	serious	none	102	105	-	SMD **0.13 SD higher** (0.27 lower to 0.52 higher)	⨁⨁⨁◯ Moderate	CRITICAL
**Theoretical scores_non-randomised studies**												
3	non-randomised studies	serious	not serious	not serious	not serious	none	299	402	-	SMD **0.42 SD higher** (0.12 higher to 0.72 higher)	⨁◯◯◯ Very low	CRITICAL

CI: confidence interval; **SMD:** standardised mean difference

C

Author(s):
Question: FC compared to TL for surgical education
Setting:
Bibliography:

Certainty assessment							№ of patients		Effect		Certainty	Importance
№ of studies	Study design	Risk of bias	Inconsistency	Indirectness	Imprecision	Other considerations	FC	TL	Relative (95% CI)	Absolute (95% CI)		
**Operational scores_randomised studies**												
2	randomised trials	not serious	not serious	not serious	not serious	none	99	98	-	SMD **0.59 SD higher** (0.3 higher to 0.88 higher)	⨁⨁⨁⨁ High	CRITICAL
**Operational scores_non-randomised studies**												
1	non-randomised studies	serious	not serious	not serious	not serious	none	64	58	-	SMD **0.51 SD higher** (0.15 higher to 0.87 higher)	⨁◯◯◯ Very low	CRITICAL

CI: confidence interval; **SMD:** standardised mean difference

Supplementary Figure 2. Certainty of evidence for total scores (A), theoretical scores (B), and operational scores (C) measured by the GRADE system.
